# Supplementary figures and images for: Low frequency weak electric fields can induce structural changes in water
Source: PLoS One. 2021 Dec 2;16(12):e0260967. doi: 10.1371/journal.pone.0260967 (PMC8639071; doi:10.1371/journal.pone.0260967)

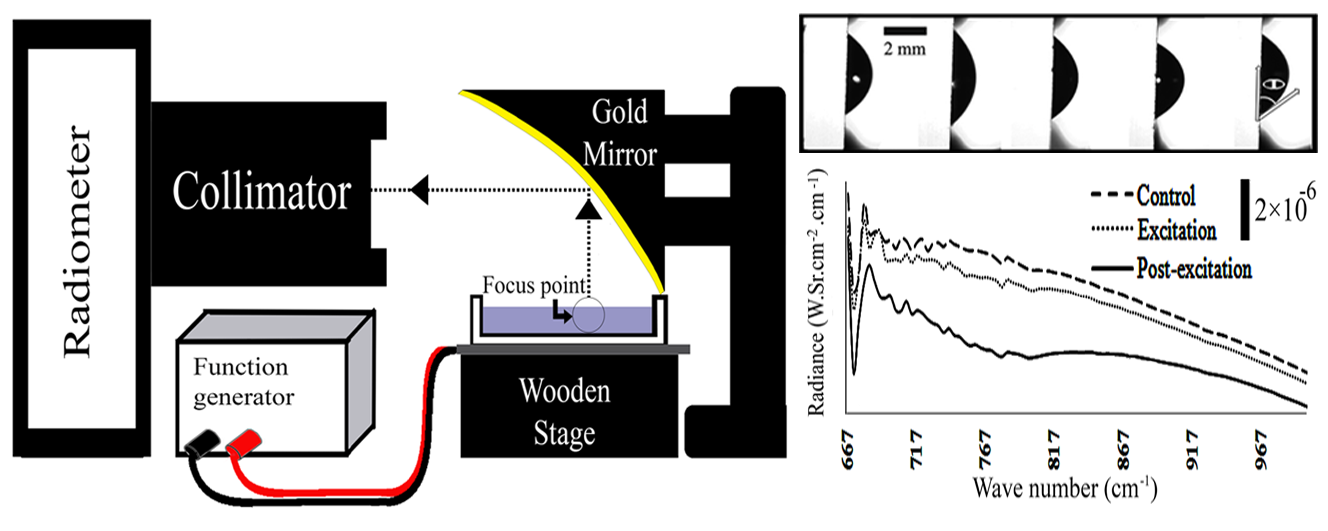

Supplement: S1 Graphical abstract — (TIF) [file pone.0260967.s002.tif]
