# Supplementary material for: Low frequency weak electric fields can induce structural changes in water
Source: PLoS One. 2021 Dec 2;16(12):e0260967. doi: 10.1371/journal.pone.0260967 (PMC8639071; doi:10.1371/journal.pone.0260967)

# PLOS ONE

## Low frequency weak electric fields can induce structural changes in water

--Manuscript Draft--

| | |
|---|---|
| **Manuscript Number:** | PONE-D-21-22146 |
| **Article Type:** | Research Article |
| **Full Title:** | Low frequency weak electric fields can induce structural changes in water |
| **Short Title:** | impact of weak electric fields on pure water |
| **Corresponding Author:** | Iman Rad, Ph.D.<br>stem cell technology research center<br>Tehran, Tehran IRAN, ISLAMIC REPUBLIC OF |
| **Keywords:** | Extremely low frequency (ELF); electric field; spectro-radiometer; radiance and water |
| **Abstract:** | Low frequency electric fields were exposed to various water samples using platinum electrodes mounted near the water surface. Responses were monitored using a spectro-radiometer and a contact-angle goniometer. Treatment of DI (deionized), EZ (Exclusion Zone), and bulk water with certain electromagnetic frequencies resulted in a drop of radiance persisting for at least half an hour. Compared to DI water, however, samples of EZ and bulk water showed lesser radiance drop. Contact-angle goniometric results confirmed that when treated with alternating electric fields (E = 600 ± 150 V/m, f = 7.8 and 1000 Hz), droplets of EZ and bulk water acquired different charges. The applied electric field interacted with EZ water only when electrodes were installed above the chamber, but not beneath. Further, when DI water interacted with an electric field applied from above (E = 600 ± 150 V/m, f = 75 Hz), its radiance profile became similar to that of EZ water. Putting these last two findings together, one can say that application of an electric field on DI water from above (E = 600 ± 150 V/m, f = 7.8 to 75 Hz) may induce a molecular ordering in DI water similar to that of EZ water. |
| **Order of Authors:** | Iman Rad, Ph.D. |
| | Rainer Stahlberg |
| | Kurt Kung |
| | Gerald H. Pollack |
| **Additional Information:** | |
| **Question** | **Response** |
| **Financial Disclosure**<br><br>Enter a financial disclosure statement that describes the sources of funding for the work included in this submission. Review the submission guidelines for detailed requirements. View published research articles from *PLOS ONE* for specific examples.<br><br>This statement is required for submission and **will appear in the published article** if the submission is accepted. Please make sure it is accurate. | The funders had no role in study design, data collection and analysis, decision to publish, or preparation of the manuscript. |

*Powered by Editorial Manager® and ProduXion Manager® from Aries Systems Corporation*

| | |
|---|---|
| **Unfunded studies**<br>Enter: *The author(s) received no specific funding for this work.*<br><br>**Funded studies**<br>Enter a statement with the following details:<br>• Initials of the authors who received each award<br>• Grant numbers awarded to each author<br>• The full name of each funder<br>• URL of each funder website<br>• Did the sponsors or funders play any role in the study design, data collection and analysis, decision to publish, or preparation of the manuscript?<br>• **NO** - Include this sentence at the end of your statement: *The funders had no role in study design, data collection and analysis, decision to publish, or preparation of the manuscript.*<br>• **YES** - Specify the role(s) played.<br><br>* typeset | |
| **Competing Interests**<br><br>Use the instructions below to enter a competing interest statement for this submission. On behalf of all authors, disclose any competing interests that could be perceived to bias this work—acknowledging all financial support and any other relevant financial or non-financial competing interests.<br><br>This statement is required for submission and **will appear in the published article** if the submission is accepted. Please make sure it is accurate and that any funding sources listed in your Funding Information later in the submission form are also declared in your Financial Disclosure statement.<br><br>View published research articles from *PLOS ONE* for specific examples. | The authors have declared that no competing interests exist. |

| | |
|---|---|
| **NO authors have competing interests**<br><br>Enter: *The authors have declared that no competing interests exist.*<br><br>**Authors with competing interests**<br><br>Enter competing interest details beginning with this statement:<br><br>*I have read the journal's policy and the authors of this manuscript have the following competing interests: [insert competing interests here]*<br><br>* typeset | |
| **Ethics Statement**<br><br>Enter an ethics statement for this submission. This statement is required if the study involved:<br><br>• Human participants<br>• Human specimens or tissue<br>• Vertebrate animals or cephalopods<br>• Vertebrate embryos or tissues<br>• Field research<br><br>Write "N/A" if the submission does not require an ethics statement.<br><br>General guidance is provided below. Consult the submission guidelines for detailed instructions. **Make sure that all information entered here is included in the Methods section of the manuscript.** | N/A |

| | |
|---|---|
| **Format for specific study types**<br><br>**Human Subject Research (involving human participants and/or tissue)**<br>• Give the name of the institutional review board or ethics committee that approved the study<br>• Include the approval number and/or a statement indicating approval of this research<br>• Indicate the form of consent obtained (written/oral) or the reason that consent was not obtained (e.g. the data were analyzed anonymously)<br><br>**Animal Research (involving vertebrate animals, embryos or tissues)**<br>• Provide the name of the Institutional Animal Care and Use Committee (IACUC) or other relevant ethics board that reviewed the study protocol, and indicate whether they approved this research or granted a formal waiver of ethical approval<br>• Include an approval number if one was obtained<br>• If the study involved *non-human primates*, add *additional details* about animal welfare and steps taken to ameliorate suffering<br>• If anesthesia, euthanasia, or any kind of animal sacrifice is part of the study, include briefly which substances and/or methods were applied<br><br>**Field Research**<br><br>Include the following details if this study involves the collection of plant, animal, or other materials from a natural setting:<br>• Field permit number<br>• Name of the institution or relevant body that granted permission | |
| **Data Availability**<br><br>Authors are required to make all data underlying the findings described fully available, without restriction, and from the time of publication. PLOS allows rare exceptions to address legal and ethical concerns. See the PLOS Data Policy and FAQ for detailed information. | Yes - all data are fully available without restriction |

Powered by Editorial Manager® and ProduXion Manager® from Aries Systems Corporation

| | |
|---|---|
| A Data Availability Statement describing where the data can be found is required at submission. Your answers to this question constitute the Data Availability Statement and **will be published in the article**, if accepted.<br><br>**Important:** Stating 'data available on request from the author' is not sufficient. If your data are only available upon request, select 'No' for the first question and explain your exceptional situation in the text box.<br><br>Do the authors confirm that all data underlying the findings described in their manuscript are fully available without restriction? | |
| **Describe where the data may be found in full sentences. If you are copying our sample text, replace any instances of XXX with the appropriate details.**<br><br>• If the data are **held or will be held in a public repository**, include URLs, accession numbers or DOIs. If this information will only be available after acceptance, indicate this by ticking the box below. For example: *All XXX files are available from the XXX database (accession number(s) XXX, XXX.)*.<br>• If the data are all contained **within the manuscript and/or Supporting Information files**, enter the following: *All relevant data are within the manuscript and its Supporting Information files.*<br>• If neither of these applies but you are able to provide **details of access elsewhere**, with or without limitations, please do so. For example:<br><br>*Data cannot be shared publicly because of [XXX]. Data are available from the XXX Institutional Data Access / Ethics Committee (contact via XXX) for researchers who meet the criteria for access to confidential data.*<br><br>*The data underlying the results presented in the study are available from (include the name of the third party* | All relevant data are within the manuscript and its Supporting Information files. |

| | |
|---|---|
| *and contact information or URL).*<br>• This text is appropriate if the data are owned by a third party and authors do not have permission to share the data.<br><br>* typeset | |
| Additional data availability information: | |

Manuscript
Click here to access/download;Manuscript;inductive draft 16.docx

# Low frequency weak electric fields can induce structural changes in water

Iman Rad[1, 2*], Rainer Stahlberg[1], Kurt Kung[1] and Gerald H. Pollack[1]

[1*] Department of Bioengineering, Box 355061, University of Washington, Seattle, Washington 98195.

[2] Current address: Stem Cell Technology Research Center, Tehran, 1997775555 Iran.

* Iman Rad

radi@u.washington.edu, rad@strc.ac.ir

Rainer Stahlberg

raista@u.washington.edu

Kurt Kung

kurt.kung@4th-phase.com

Gerald H. Pollack

ghp@u.washington.edu

## Abstract

Low frequency electric fields were exposed to various water samples using platinum electrodes mounted near the water surface. Responses were monitored using a spectro-radiometer and a contact-angle goniometer. Treatment of DI (deionized), EZ (Exclusion Zone), and bulk water with certain electromagnetic frequencies resulted in a drop of radiance persisting for at least half an hour. Compared to DI water, however, samples of EZ and bulk water showed lesser radiance drop. Contact-angle goniometric results confirmed that when treated with alternating electric fields (E = 600 ± 150 V/m, *f* = 7.8 and 1000 Hz), droplets of EZ and bulk water acquired different charges. The applied electric field interacted with EZ water only when electrodes were installed above the chamber, but not beneath. Further, when DI water interacted with an electric field applied from above (E = 600 ± 150 V/m, *f* = 75 Hz), its radiance profile became similar to that of EZ water. Putting these last two findings together, one can say that application of an electric field on DI water from above (E = 600 ± 150 V/m, *f* = 7.8 to 75 Hz) may induce a molecular ordering in DI water similar to that of EZ water.

**Keywords:** Extremely low frequency (ELF), electric field, spectro-radiometer, radiance and water.

## 1. Introduction

In a previous report [1], we used a spectro-radiometer and an infrared camera to monitor the impact of applied electric currents in water. That work showed that application of extremely low frequency alternating currents induced structural changes in water, which were also confirmed by IR radiance spectra [1, 2]. Structural changes may also be reflected in changes of temperature: proximity of water to charged surfaces can trigger temperature drop, or even freezing [3, 4].

One of the challenges of the previous study was our inability to rule out the intervening impact of electrolysis during the application of electric current, i.e., when electrodes were immersed in water [5]. Therefore, in the current study, we attempted to determine whether external electric fields, i.e., with electrodes outside the water, and with relatively lower amplitudes, might cause similar structural changes.

Field strengths of $10^7$-$10^8$ V/m exist in nature, e.g. across the membrane of a resting cell with ~ 1-5 nm thickness [6, 7]. Theoretical predictions suggest that field strengths of $10^7$-$10^9$ V/m considerably diminish translational entropy of confined water [3, 4], which results in a temperature drop, or even freezing. An electric field with maximal amplitude of only ~ 750 V/m, as used in the current study, is not expected to make non-confined water freeze, but may diminish its temperature to some extent.

Here, we applied external electric fields on non-confined water samples to determine if any of those fields can cause a drop of temperature, or radiance. We also discuss potential mechanisms that may explain the observed results.

# 2. Experimental section

## 2.1. Experimental design

Three types of water were used in this study: DI (deionized), EZ (exclusion-zone) and bulk water. EZ and bulk waters, acquired from 4$^{th}$-Phase Inc., were prepared by passing DI water through a Nafion tube (Perma Pure LLC), and extracting each of the two phases. Details of separation appear in the first figure of US7819259B2 patent [8].

Essentially, a Nafion tube was mounted between electrodes. EZ and bulk waters were formed in place, around the 6-mm long Nafion tube. After 10 minutes, an annular EZ layer commonly grew to a width of approximately 500 µm [9]. By examining the behavior of each of the above-mentioned waters directly in the Nafion setup, features of the EZ, as well as the bulk water beyond, could be directly assessed.

Since EZ formation next to Nafion is temperature dependent [10], after EZ and bulk waters were collected, we used a thermal stage to set and maintain the temperature. Experiments were run at 293, 283 and 273 K. Thermal control was set via a temperature control module (Omegaette PV), as previously explained in detail [11].

## 2.2. Measurement setup

All experiments were conducted with purified water inside a shallow rectangular polypropylene dish (4.5 cm wide, 2.5 cm long and 0.5 cm high). The DI water was HPLC grade, 18.2 $M\Omega.cm^{-1}$, obtained from a standard water-purification system (Diamond TII, Barnstead). The chamber was filled with three milliliters of either DI, EZ, or bulk water for different experiments. Before the experiment began, the chamber was filled, and left for 30 minutes for the water to become thermally stable. Then, exposure to the external electric field began. Water was exposed to alternating electric fields generated by a function generator (SRS, Model DS335) at a series of frequencies. The applied voltage was 6 ± 1.5 V, which created electric fields in the range of 450 – 750 V/m. This range is defined mathematically as a vector field associated with the distance between electrodes in a vacuum, and does not reflect the time that power is delivered. Exposure time extended over 30 minutes. This particular range of electric field strength was chosen since application of electric fields with amplitude of less than 750 V/m does not apparently freeze bulk

water [4]. The field was created using two electrodes, installed either beneath or above the water-containing chamber (Figure 1A).

Platinum wires (Sigma-Aldrich, CAS: 7440-06-4, 5-cm length, 2-mm diameter) were used as electrodes. They were installed at a distance of 1 cm from one another, in the horizontal plane. The electrode pairs were set either 1 mm above the container-water surface or 1 mm beneath (below the bottom of the water container - Figure 1B, C).

The electrical energy needed for complete electrolysis of pure water in this apparatus, total conversion of water to $H_2$ and $O_2$, can be calculated to lie between 1.3 - 13 Watt-hours. We estimated the possible electrolysis in this study to be almost $10^9$ times lower than that, i.e., between 0.8 - 4.38 × $10^{-9}$ Watt-hours.

## 2.3. IR-radiance recording setup

An MR170 spectro-radiometer (ABB Measurement & Analytics) was used to record the emission spectra in the wavelength range of 2 - 15 µm (5000 – 667 $cm^{-1}$) by the device's Fourier transforming software (FTSW500 Version 1.06).

The experimental setup and its software settings are explained in detail in a previous report [1]. Reproducibility of the measurement is correlated to the recorded noise: The higher the noise amplitude, the lower the expected reproducibility. In our experiments, calibration residuals were 10 to 20 times lower than the radiance amplitude, and could therefore be ignored. Radiance is expressed, in standard SI units, as watts per steradian per square centimeter per centimeter ($W \cdot Sr^{-1} \cdot cm^{-2} \cdot cm^{-1}$).

The schematic design of the experiment is shown in Figure 1. To record reproducible radiance values from spots of 1 - 2 mm diameter, we focused the IR emission using a concave gold

mirror. The mirror's focus area is a circle of 2 mm diameter. The mirror reflected the emitted infrared radiation from the water surface into the spectro-radiometer's collimator tube.

### 2.3.1 IR-radiance of EZ and bulk water in the presence of Nafion

Besides use of the apparatus described above, we also wished to determine whether the interfacial water situated near the surface of a Nafion tube could be radiometrically differentiated from the bulk water, the latter situated at some distance away. By focusing the radiometer's gold mirror on the desired area, we could make this determination. To this aim, we focused on the area between two walls of the Nafion tube that encompasses two EZs on the edges and bulk in between (i.e. the entire inside of the tube - Figure 1C). To measure bulk-water radiance, we placed the focus of the mirror 2 cm away from the Nafion wall. The mirror was similarly positioned for measuring the radiance of control water, but without the Nafion tube.

### 2.3.2 IR-radiance of EZ, bulk, and DI water after treatment with electric field

The properties of EZ and bulk water around the Nafion tube have been explored in previous studies [12, 13]. Here, we measured the impact of the electric-field on the EZ or bulk water. As first step, we compared the radiances of the field-treated EZ and bulk waters, with the corresponding untreated radiances as control. Since the electrodes were outside the water, they remained remote from the area of measurement.

## 2.4. Contact-angle measurement

The spreading of a water droplet on a surface is routinely measured by the "contact angle" method. Contact-angle (goniometric) measurements are useful in the evaluation of surface macroscopic properties, such as surface energy and wettability [14]. Aqueous solutions in

contact with surfaces experience strong Coulomb forces at their interfaces due to the charge interaction between the solution and surface [15-17]. Once the surface charge is known, the interfacial energy of such liquids facing solid surfaces can be estimated according to the measured static contact angle (θ) [18]. Therefore, knowing the charge of the surface in contact with the solution droplet, one can estimate the overall charge distribution of the droplet.

In order to monitor the spreading of a water droplet on positively and negatively charged surfaces, Teflon, Plexiglas, and glass surfaces were used. The aforementioned surfaces have static electric potentials of 7000 ± 500, -8000 ± 500 and 15 ± 10 V/m, respectively, which marks them as positively, negatively and almost neutrally charged surfaces that result in corresponding contact angles of 95.3, 66.7 and 40 degrees, respectively, in a spreading DI water droplet [14]. Static electric potential of the surfaces was measured by Static Locater/Meter Model 9000 (PN 90930-10001) non-contacting voltmeter.

To measure contact angle (θ), a CCD camera was set beside a horizontally positioned sheet with the droplet in the middle. The captured side view of each water droplet was recorded, and then analyzed. The contact angle "θ" of each water droplet was calculated using Image J software. Contact angle measurements were repeated five times and averaged.

### 2.5 Data analysis

Raw spectral data emerge as the average of 100 scans, which took 10-20 seconds to acquire, as described previously [1]. Both radiometric and contact angle measurements were statistically analyzed using one-way ANOVA.

## 3. Results

We used a spectro-radiomenter to measure the microscopic structural variations of water, while macroscopic charge distribution was assessed via contact-angle measurements. It was hoped that the results of these combined methods could bridge the gap between microscopic and macroscopic behaviors of water droplets.

In order to first rule out the possibility that radiation from the electrodes themselves might interfere with the radiometric measurements, an electrical potential was applied between the electrodes, installed either beneath or above the empty chamber. Voltages were applied for 30 minutes, while radiance was measured both during that period and for 30 minutes after the voltage application had ceased. Radiometric measurements of the electrodes alone (no water sample was involved) showed that when voltage was applied, no significant electrode radiance was observed, which implies that voltage application by the electrodes should not interfere with the radiometric measurements.

We then treated the water with electric fields of various frequencies. Fields were turned on for 30-minute periods. Radiometric measurements were made during the 30-minute period and for another 30 minutes thereafter. The latter period is referred to as the “post-excitation” phase. The entire time course, including excitation and post-excitation, was limited to 60 minutes to avoid excessive evaporation, which would diminish chamber-water volume and hence potentially affect the results. Each radiometric experiment was repeated three to five times to ensure reproducibility.

## 3.1. Application of electric fields to DI water

These experiments focused on standard DI water. The DI water alone was subjected to 30 minutes of “excitation”. The “control” water received no excitation.

When frequencies of 0, 4, 11, 16, 50, 75, 100 and $10^5$ Hz were applied from beneath, the radiance of DI water remained unchanged during control, excitation, and post-excitation periods. However, similar application of 7.8 and $10^3$ Hz (1 kHz) caused a reduction of water radiance relative to the control, at specific wave numbers. They ranged from 667 – 1300 and 667 – 1000 $cm^{-1}$, respectively (Figure 2$A_1$, $A_2$). The reduction was observed during the excitation as well as post-excitation periods (Figure 2).

In another set of experiments, electric fields with frequency of 0, 4, 7.8, 11, 16, 50 and $10^5$ Hz were applied to DI water from above. Results showed no change in radiance during excitation and post-excitation periods. However, application of 75, $10^2$ and $10^3$ Hz frequencies from above dropped the water radiance, both during excitation and post-excitation periods (Figure 3$A_1$ to $A_3$).

## 3.2. Application of electric field on bulk water

To obtain "bulk" water, we used Nafion tubes. The water that was collected from just inside the Nafion-tube wall (within 100 μm proximity) was taken as EZ water, while the water passing through the core of the tube (more than 500 μm away from the wall) was considered bulk water.

Frequencies were chosen based on earlier observations. Since application of electric fields to DI water showed a substantial radiance drop at frequencies of 7.8, 75, $10^2$ and $10^3$ Hz, these four frequencies were chosen for testing on preparations of "EZ" and "bulk" water.

Application of electric field with frequency of 7.8 Hz. on bulk water either from above or beneath had no impact on the water radiance. When the applied frequency was increased to 75 Hz, the excitation and post-excitation radiance dropped in the spectral region 667 – 1000 $cm^{-1}$. A similar drop was observed when a frequency of $10^3$ Hz applied from above (Figure 4A, B).

## 3.3. Application of electric field to EZ water

Samples of "EZ water" were prepared the day prior to the experiment. The "EZ" samples are never completely pure EZ, and may be partly mixed with some bulk water.

Electric fields with frequencies of 7.8, 75, $10^2$ and $10^3$ Hz were applied to EZ water from beneath and above, independently. Only when the frequency of 7.8 Hz was applied from above did the radiance drop during excitation and post-excitation periods (Figure 5). Other treatments produced no observable change.

## 3.4. Effect of temperature on radiance

Positioning of a Nafion tube in DI water leads to the formation of EZ and bulk water (Figure 1C). With the tube in place, we measured radiance of EZ water around the Nafion tube, and also the bulk water — at different temperatures. By this method, the impact of temperature on the radiance of EZ and bulk water could be differentiated more decisively. For example, at 293 K, in the spectral region of 667 - 1000 $cm^{-1}$ the radiance of both EZ and bulk water were significantly lower than that of DI water (Figure 6A, B). In other spectral regions, the radiance of the two did not differ.

When the water temperature diminished to 283 °K, the radiance of EZ water at 667 – 2000 $cm^{-1}$ was lower than that of bulk and DI waters (Figure 6C, $C_1$, $C_2$ and $C_3$). Moreover, with further water-temperature decrease to 273 K, the radiance of control, EZ and bulk waters became non-differentiable. Therefore, EZ and bulk water are more profoundly differentiable at higher temperature than a lower temperature.

## 3.5. Variation of charge in different treated water droplets

Here we used 50 μL droplets of EZ, bulk, and DI water. After being treated with 7.8 Hz and 1000 Hz electric fields, EZ and bulk water droplets of the same volume were tested by contact-angle measurement. Measurement of each type of water droplet was repeated five times.

Droplets of untreated water, EZ and bulk, were considered as "EZ control" and "bulk control", respectively. Experimental samples were treated, respectively, with electric fields of 7.8 Hz ("EZ 7.8 Hz") and 1000 Hz ("bulk 1000 Hz"), (Figure 7A). As mentioned in section 3.2., frequencies were chosen based on earlier observations.

Measurement of the contact angle "θ", acquired from different types of water droplets on the neutrally charged surface of the glass, showed no notable differences among "EZ control", "EZ 7.8 Hz", "Bulk control" and "Bulk 1000 Hz" (Figure 7B).

On the positively and negatively charged surfaces, such as Teflon and Plexiglas, comparison of sitting water droplets showed that "Bulk control" droplet made a larger contact angle "θ" compared to the "Bulk 1000" droplet (Figure 7C).

# 4. Discussion

Application of electric fields of certain frequencies diminished water radiance. The fields, of magnitude 600 ± 150 V/m, near the electrodes were applied either from above or beneath the water-containing chamber. Deionized water samples were treated with frequencies of 0, 4, 7.8, 11, 16, 50, 75, $10^2$, $10^3$ and $10^5$ Hz. (Note that the frequency of 7.8 Hz, the Schumann frequency, was used instead of 8 Hz, since this frequency is reported to have a role in

transduction of biological information [2, 19, 20]). During control, excitation, and post-excitation periods, we assessed potential structural changes by measuring the water's IR radiance. Besides, EZ and bulk water droplets that were treated with electric fields also showed different radiance and charge distribution. In the following section, we will discuss electric field impacts in detail.

## 4.1. Radiometric findings

Depending on the position of the electrodes, beneath vs. above, and the frequency of the applied electric field, water samples' radiance changed differently. For instance, when the electric field ($E = 600 \pm 150$ V/m) was applied to DI water from above with frequencies of 75, $10^2$ or $10^3$ Hz, or to bulk water with frequency of $10^3$ Hz, water radiance diminished within the 677 – 1000 $cm^{-1}$ range. Similar impact was observed upon application of the electric field from beneath, with frequencies of 7.8 and $10^3$ Hz on DI water, or with frequencies of 75 and 7.8 Hz on bulk and EZ waters, respectively (Figures 2, 3 and 5). Other frequencies produced no significant changes in radiance.

Thus, application of electric fields from beneath imposed a radiance-diminishing impact on all water-sample types, while application of electric field from above modified only DI and bulk-water radiance, but not EZ radiance.

Since both DI and bulk waters showed diminished radiance independent of field direction, while EZ water showed diminished radiance only when the field was applied from above (Figure 5), it appears that EZ water may be more reluctant to acquire further structural modification after exposure to the electric field. A possible interpretation is that EZ water is more stable than DI or bulk water. Since it is already ordered, any additional ordering caused by electric-field application may be difficult to achieve.

When liquid water is subject to an inhomogeneous electric field ($\nabla^2 E_a \approx 10^{10}$ V/m$^2$), enhancement of the vibrational collective modes, hindered rotational freedom of long range dipole–dipole interactions, and retardation of heat flow occurs, which eventually generates a chemical potential gradient in electrified water [21]. In other words, liquid water stressed by an electric field, exhibits primary signs of phase transition throughout the entire volume of liquid [22]. Previously, it was reported that water molecules tend to get ordered because of diminution in their translational entropy [1, 23, 24]. Diminished translational entropy is also detectable as a sharpened peak in radiometric measurements around 600 cm$^{-1}$, and a temperature drop from 300 °K to 240 °K [25]. In this study, application of an electric field resulted in diminished translational entropy and temperature drop in DI and EZ water (Figure 2, 3, 5 and 6), although not in bulk water (Figure 4).

## 4.2. Insights from contact-angle measurements

Contact-angle measurement comes with the expectation that positively charged water droplets have the highest contact angle on a positively charged surface, and the smallest contact angle on a negatively charged surface, and vice-versa for negatively charged droplets. Thus, depending on the charge of surface and water sample, a droplet acquires a specific contact angle "θ" [26]. If both the water droplet and the surface have same electrical charge, θ will change from 90 degrees toward higher angles due to repulsion. Otherwise, the θ angle will drop from 90 degree toward lower angles.

With this understanding, it appears that both "bulk control" and "bulk 1000 Hz" droplets had less positive charge content than both the "EZ control" and "EZ 7.8 Hz" droplets (Figure 7B). The "bulk control" and "bulk 1000 Hz" droplets did not show any difference when they sat on a glass

surface (Figure 7B). However, when both “bulk control” and “bulk 1000 Hz” sat on a Teflon surface, it appeared that the “bulk control” droplet was more positively charged than “bulk 1000 Hz” droplet (Figure 7C).

Putting all the contact-angle results together, we can conclude that bulk and EZ water droplets have different charge content, making them positively or negatively charged with respect to one another. Treatment of EZ water with an electric field ($f$ = 7.8 Hz) makes the droplet more positive, while application of electric field on bulk water ($f$ = 1000 Hz) makes it more negative. Hence, these fields appear to confer differing charges to water.

## Conclusion

Treatment of EZ and bulk-water droplets with alternating electric fields altered their charge. Mainly, they appeared to gain positive charge, in the following order: bulk-control < bulk-1000 Hz < EZ-control, DI and EZ-7.8 Hz. Putting the radiometric and contact-angle goniometric results together led us to conclude that EZ water has higher structural stability and greater tendency to absorb positive charge than DI and bulk water.

On the other hand, our principal results show that application of an electric field from above, with frequency of 75 Hz on DI water, changes the water’s molecular structure toward EZ water, while other frequencies had no such impact. That frequency would result in “relatively ordered water”, a signature feature of EZ water.

## Acknowledgment

We thank the Software AG foundation (SAGST) for the support of this work.

***Figure captions***

***Figure 1:*** *Schematic views of measurement setup. (A) Lateral view of spectro-radiometer and water chamber, the latter resting on a non-conductive wooden stage. Arrowheads denote the direction of travel of light from chamber to collimator. Red and black wires represent positive and negative poles, respectively. The schematic representation of the golden mirror's focus area, from lateral view, is confined to a circle. (B) Lateral view of electrodes that were installed beneath (upper panel) or above (lower panel) the water chamber. (C) Special case, in which EZ and bulk waters around Nafion tube were studied radiometerically, while neither an excitation nor an electrode was involved. Drawing not to scale.*

***Figure 2:*** *Radiance spectra of DI water during and after application of low-frequency electric field when electrodes were installed beneath the chamber. The amplitude scale is shown by a vertical bar at the right top of the figure, and the vertical axes have the same scale. Horizontal arrow denotes a region that has considerably different amplitude compared to control.*

***Figure 3:*** *Radiance spectra of DI water, obtained when electric field was applied from above the chamber.*

***Figure 4:*** *Radiance of "bulk water" during and after exposure to electric field. The measurements were made with electrodes placed either beneath (A) or above (B) the chamber.*

***Figure 5:*** *EZ water radiance profile during and after exposure to 7.8 Hz electric field. Electrodes placed above the chamber.*

***Figure 6:*** *Radiance of water during and after exposure to Nafion tube. (A) Schematic top view of water chamber when a 6-millimeter long Nafion tube was placed at the center of the water chamber. The pale area around the tube represents EZ water. "Control" represents the*

*measurement taken before the Nafion tube had been placed in the water. (B) Radiance of EZ and bulk water at room temperature (293 K). (C) Radiance of EZ and bulk water at 283 K is shown with higher magnification at different wave-number regions ($C_1$ to $C_3$).*

***Figure 7:*** *Contact-angle measurement of five droplet types. (A) Comparision of droplet shapes and curvatures on uncharged, positively charged, and negatively charged surfaces. (B) Comparison of measured contact angle of water droplets. The stars represent the significant differences among cases or groups.*

## References


1. Rad, I. and G.H. Pollack, *Cooling of Pure Water at Room Temperature by Weak Electric Currents.* The Journal of Physical Chemistry B, 2018. **122**(31): p. 7711-7717.
2. Rad, I. and K. Jalali, *Electronic transmission of antibacterial property into water at extremely low frequency range: A preliminary study*. The Journal of Alternative and Complementary Medicine, 2018. **24**(5): p. 431-438.
3. Choi, E.-M., et al., *Freezing transition of interfacial water at room temperature under electric fields.* Physical review letters, 2005. **95**(8): p. 085701.
4. Montagnier, L., et al., *Water Bridging Dynamics of Polymerase Chain Reaction in the Gauge Theory Paradigm of Quantum Fields.* Water, 2017. **9**(5): p. 339.
5. Fuchs, E.C., D. Yntema, and J.J.J.o.P.D.A.P. Woisetschläger, *Raman spectroscopy and shadowgraph visualization of excess protons in high-voltage electrolysis of pure water.* 2019. **52**(36): p. 365302.

6. Rad, I., et al., *Novel aspects of spinal cord evoked potentials (SCEPs) in the evaluation of dorso-ventral and lateral mechanical impacts on the spinal cord.* Journal of neural engineering, 2014. **12**(1): p. 016004.

7. Nazari, H., et al., *Electrical stimulation induces differentiation of human cardiosphere‑derived cells (hCDCs) to committed cardiomyocyte.* Molecular and cellular biochemistry, 2020.

8. Pollack, G.H., *Separating components of aqueous mixtures, suspensions, and solutions*. 2010, Google Patents.

9. Rohani, M. and G.H. Pollack, *Flow through horizontal tubes submerged in water in the absence of a pressure gradient: mechanistic considerations.* Langmuir, 2013. **29**(22): p. 6556-6561.

10. Chai, B., H. Yoo, and G.H. Pollack, *Effect of radiant energy on near-surface water.* The Journal of Physical Chemistry B, 2009. **113**(42): p. 13953-13958.

11. Stahlberg, R., H. Yoo, and G.H. Pollack, *Origin of the infra-red emission peak in freezing water.* Indian Journal of Physics, 2019. **93**(2): p. 221-227.

12. Yoo, H., R. Paranji, and G.H. Pollack, *Impact of hydrophilic surfaces on interfacial water dynamics probed with NMR spectroscopy.* The journal of physical chemistry letters, 2011. **2**(6): p. 532-536.

13. Elia, V., et al., *Experimental study of physicochemical changes in water by iterative contact with hydrophilic polymers: A comparison between Cellulose and Nafion.* Journal of Molecular Liquids, 2018. **268**: p. 598-609.

14. Lamour, G., et al., *Contact angle measurements using a simplified experimental setup.* Journal of chemical education, 2010. **87**(12): p. 1403-1407.

15. Alavijeh, M.S., et al., *Antibacterial properties of ferrimagnetic and superparamagnetic nanoparticles: a comparative study.* Journal of Mechanical Science and Technology, 2021: p. 1-7.

16. Alinezhad-Bermi, S., et al., *Hyperosmolarity benefits cartilage regeneration by enhancing expression of chondrogenic markers and reducing inflammatory markers.* In Vitro Cellular & Developmental Biology-Animal, 2021: p. 1-10.

17. Alavijeh, M.S., et al., *Cobalt ferrite decoration of molybdenum disulfide nanosheets; development of a nanocomposite-mediated hyperthermia method.* Journal of Mechanical Science and Technology, 2021: p. 1-7.

18. Janssens-Maenhout, G. and T. Schulenberg, *An alternative description of the interfacial energy of a liquid in contact with a solid.* Journal of colloid and interface science, 2003. **257**(1): p. 141-153.

19. Rodríguez-Camacho, J., et al. *Application of the Singular Spectrum Analysis to the time variations of the amplitude of Schumann resonance measurements*. in *2020 28th European Signal Processing Conference (EUSIPCO)*. 2021. IEEE.

20. Nazari, H., et al., *Electrical stimulation induces differentiation of human cardiosphere-derived cells (hCDCs) to committed cardiomyocyte.* Molecular and cellular biochemistry, 2020. **470**: p. 29-39.

21. Wexler, A.D., et al., *Non-equilibrium thermodynamics and collective vibrational modes of liquid water in an inhomogeneous electric field.* 2016. **18**(24): p. 16281-16292.

22. Fuchs, E.C., et al., *Electrically Induced Liquid–Liquid Phase Transition in a Floating Water Bridge Identified by Refractive Index Variations.* 2021. **13**(5): p. 602.

23. Schmit, J.D., M. Feric, and M. Dundr, *How Hierarchical Interactions Make Membraneless Organelles Tick Like Clockwork.* Trends in Biochemical Sciences, 2021.

24. Mensink, L.I., S. de Beer, and J.H. Snoeijer, *The role of entropy in wetting of polymer brushes.* Soft Matter, 2021.

25. Zasetsky, A.Y., A.F. Khalizov, and J.J. Sloan, *Local order and dynamics in supercooled water: A study by IR spectroscopy and molecular dynamic simulations.* The Journal of chemical physics, 2004. **121**(14): p. 6941-6947.

26. Ismail, M.F., et al., *Prediction of surface charge properties on the basis of contact angle titration models.* Materials Chemistry and Physics, 2021. **258**: p. 123933.

graphical abstract

Click here to access/download;Figure;graphical abstract.tif

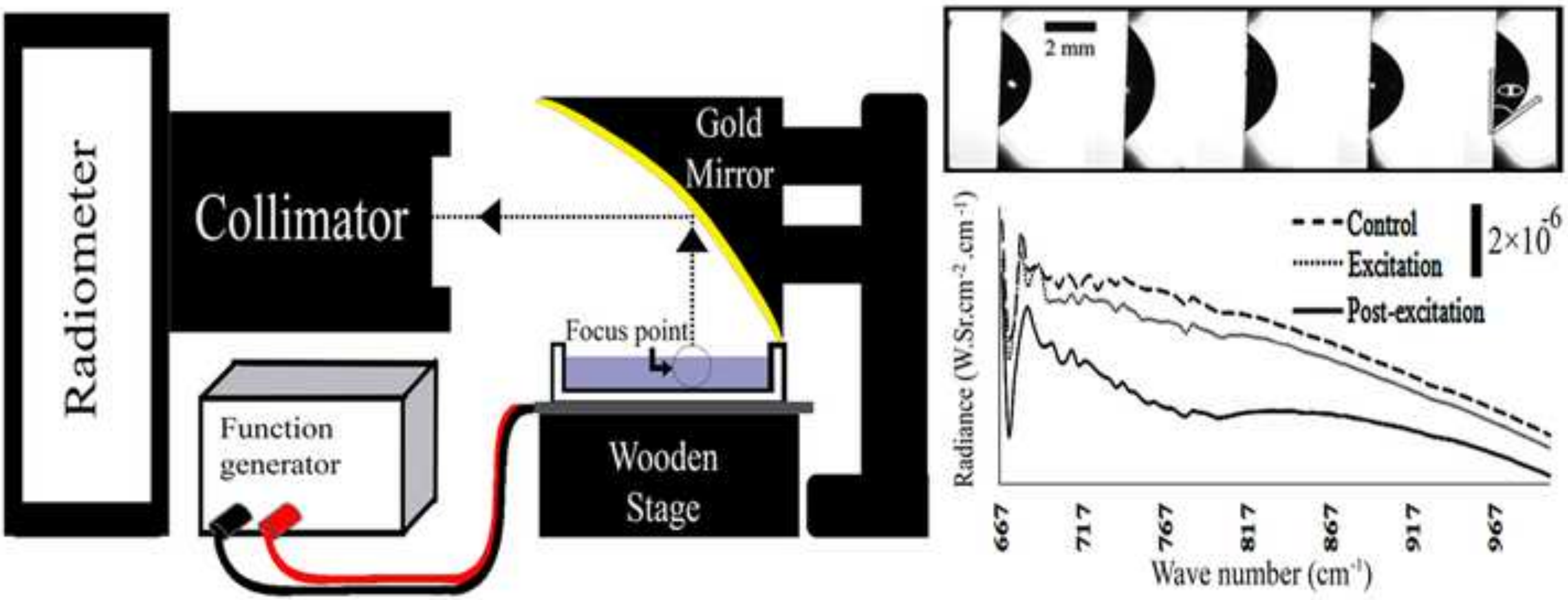

Fig. 1

Click here to access/download;Figure;Fig 1.tif

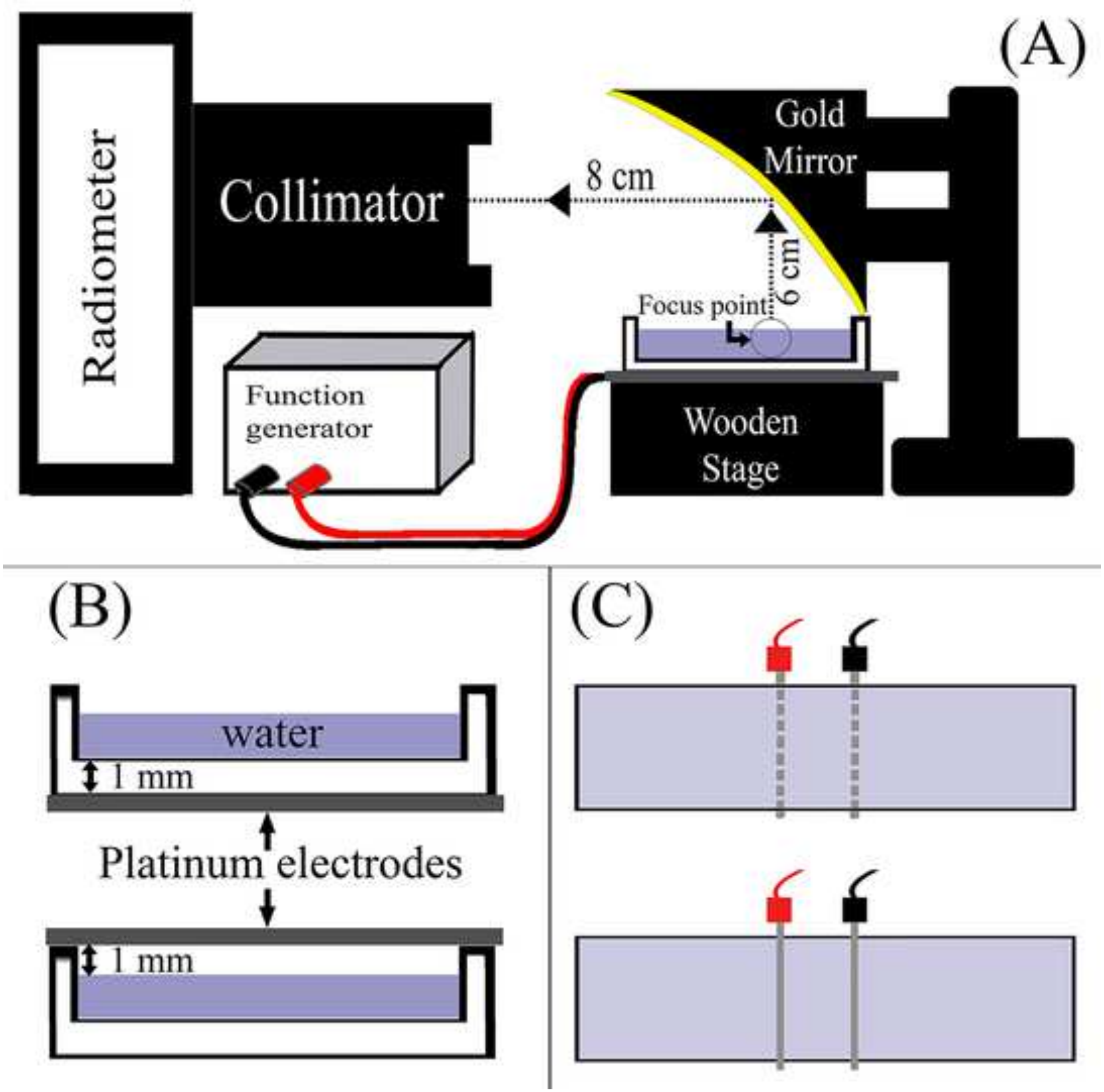

Fig. 2

Click here to access/download;Figure;Fig 2.tif

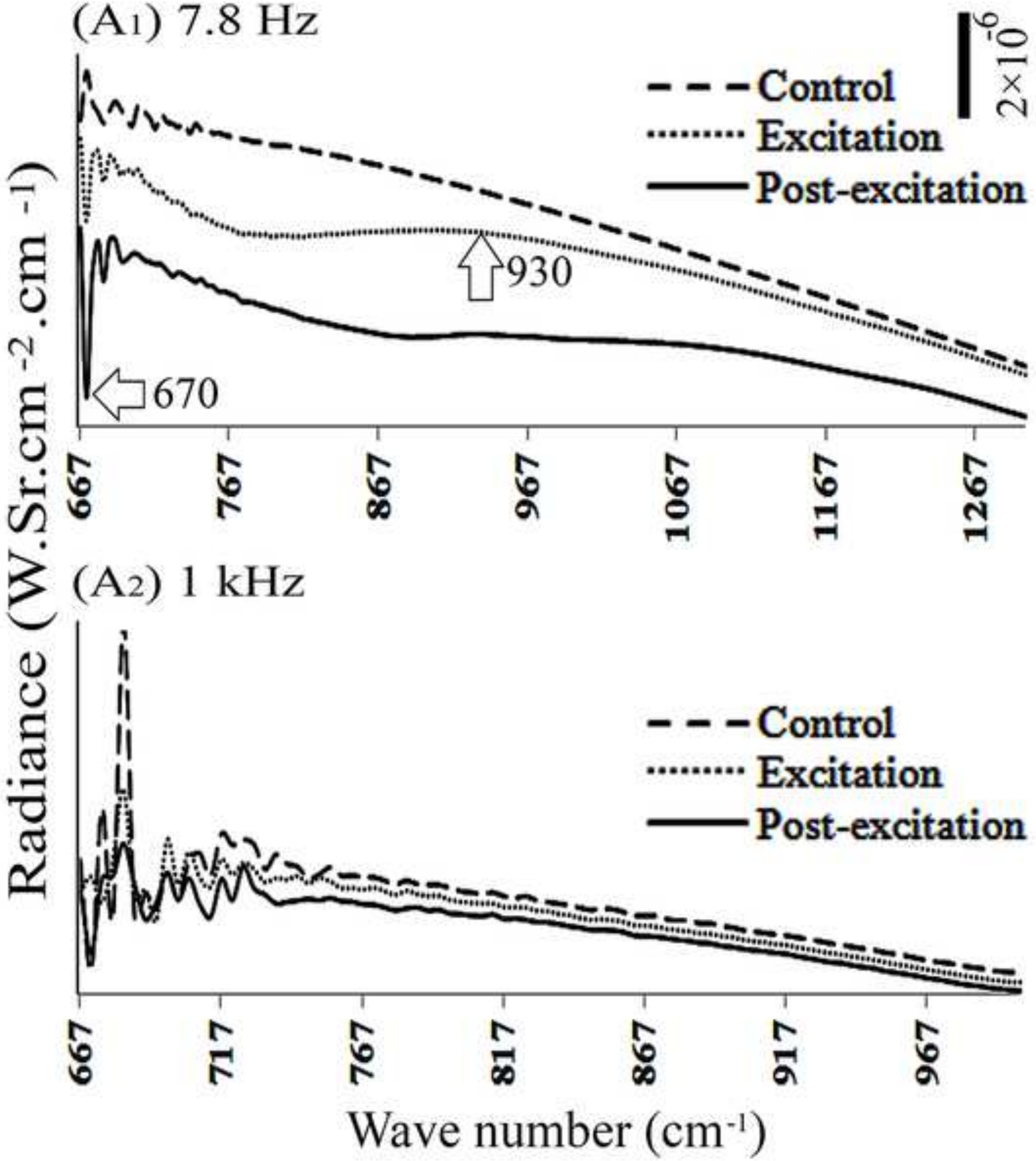

Click here to access/download;Figure;Fig 3.tif

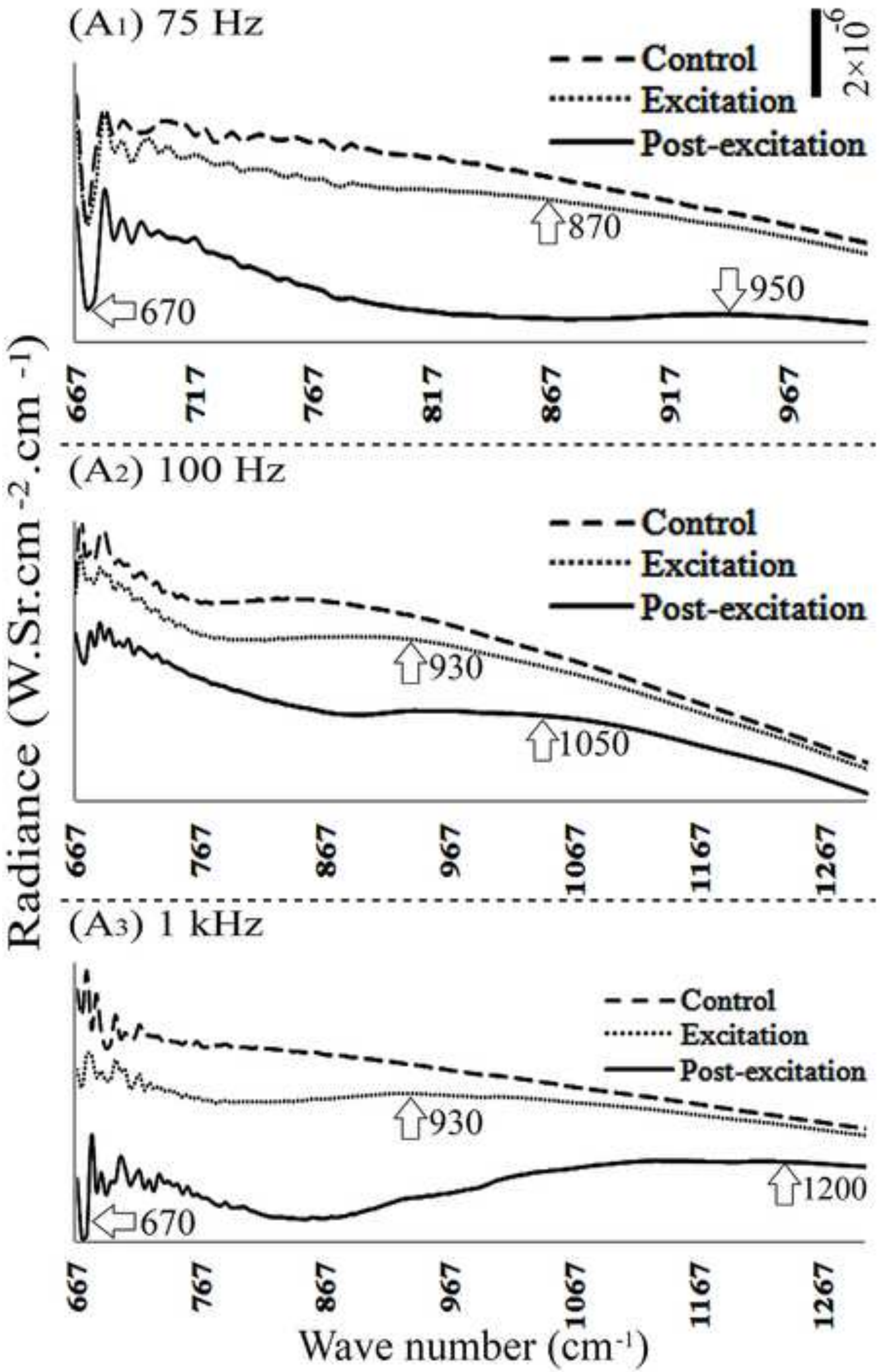

Fig. 4

Click here to access/download;Figure;Fig 4.tif

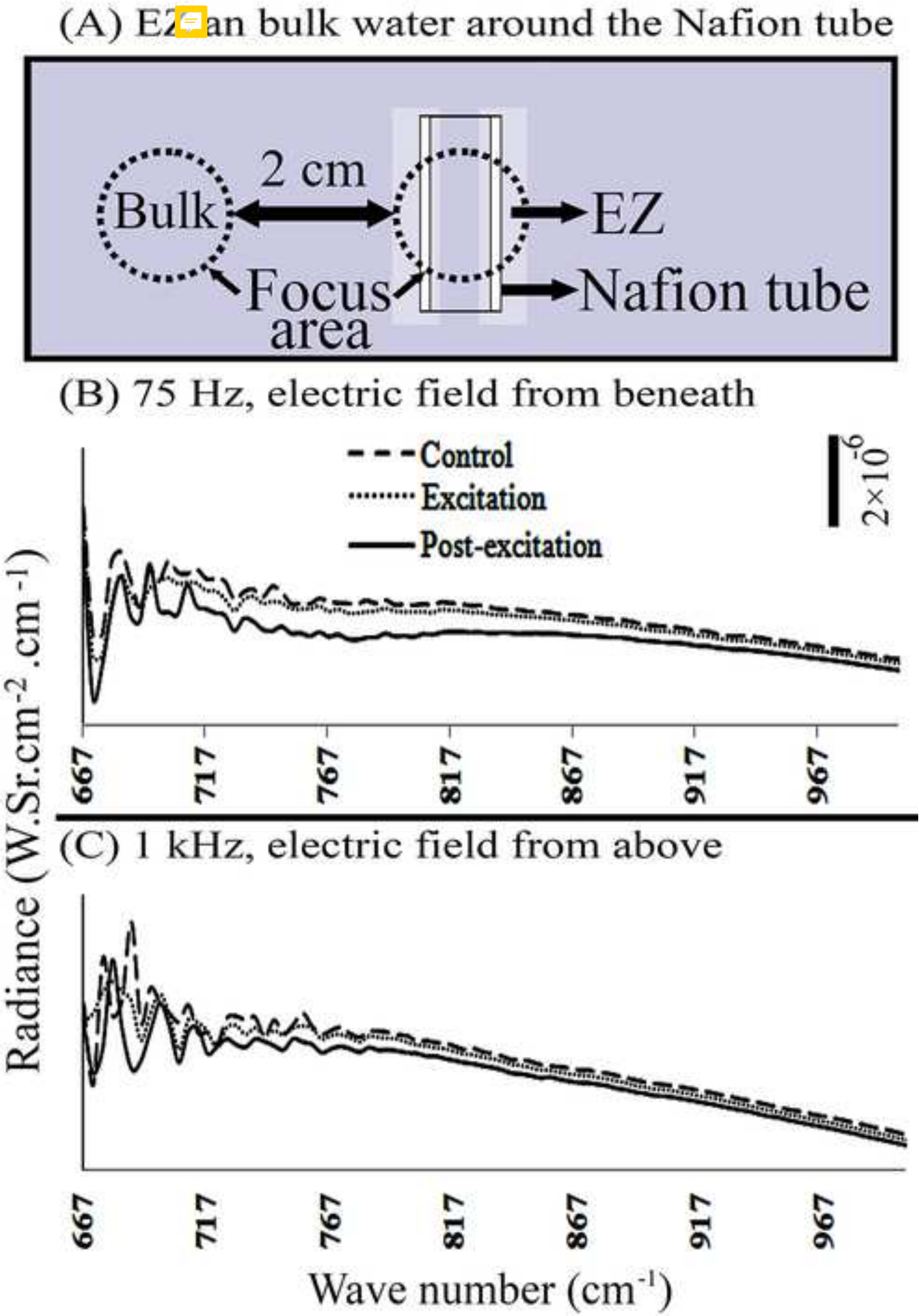

Fig. 5

Click here to access/download;Figure;Fig 5.tif

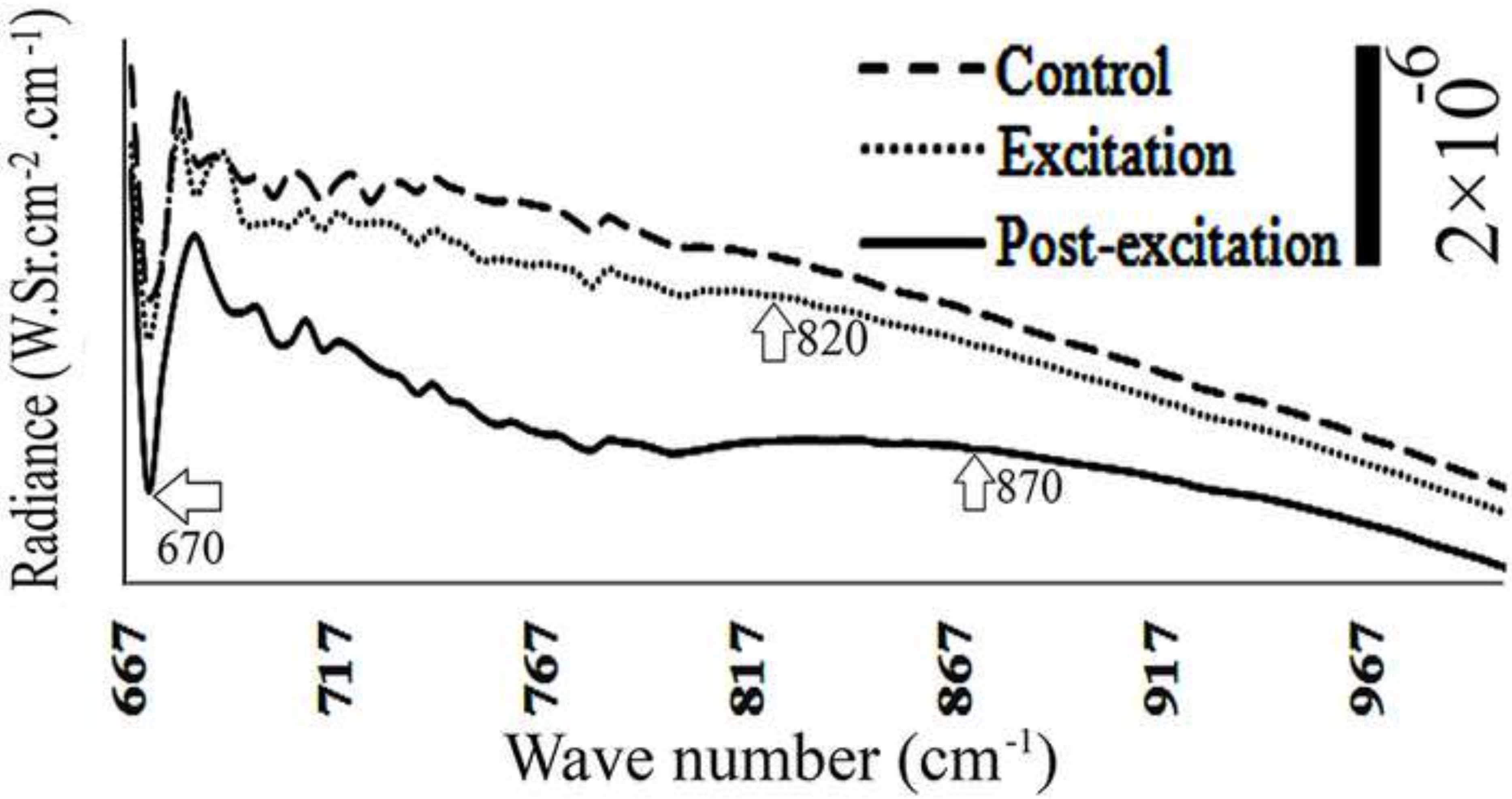

Fig. 6

Click here to access/download;Figure;Fig 6.tif

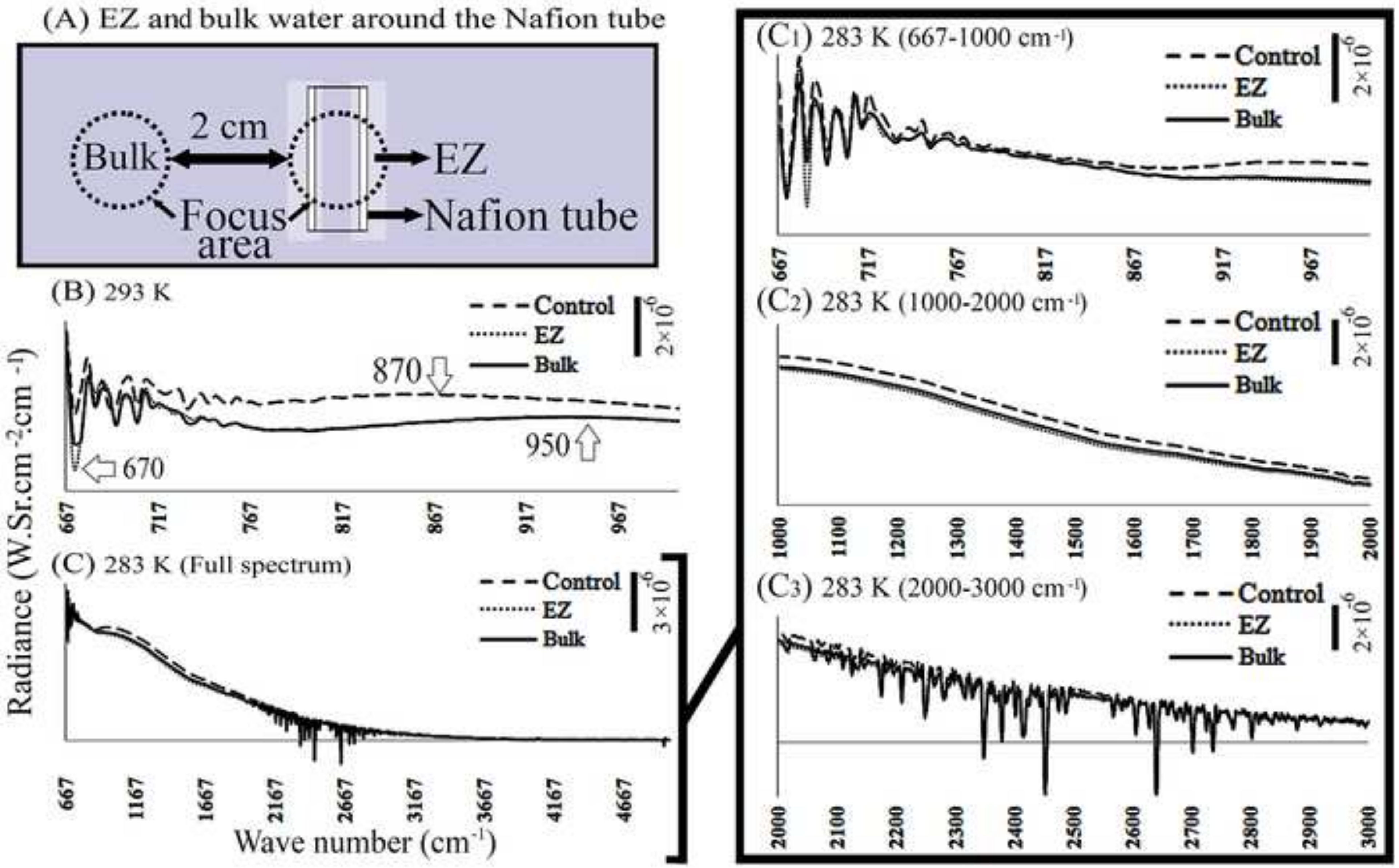

Click here to access/download;Figure;Fig 7.tif

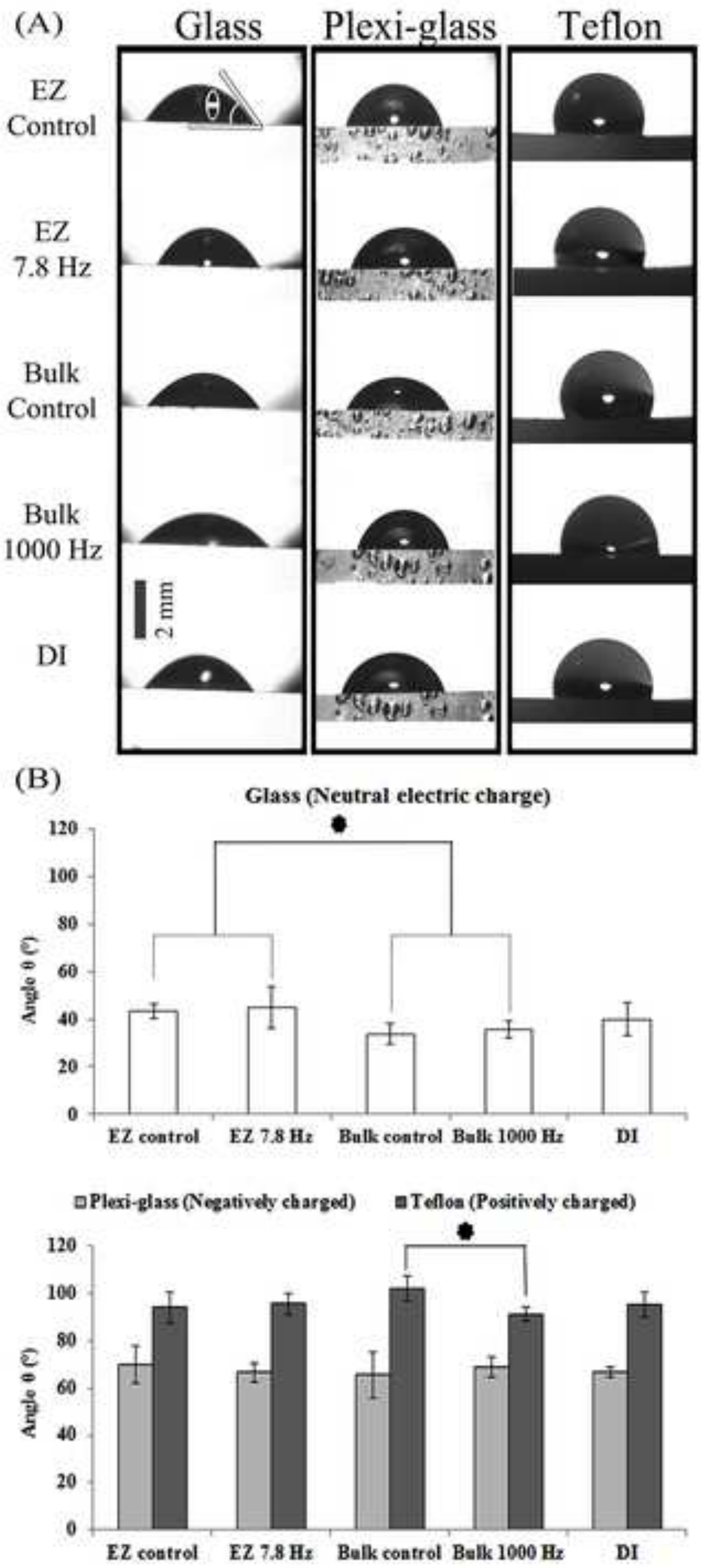

Supplement: Attachment — Submitted filename: PONE-D-21-22146_reviewer_Low frequency weak electric fields can induce structural changes in water_Rad.pdf [file pone.0260967.s003.pdf]
